# Heavy metals in wild and cultured shrimp, supplied feeds, and their habitats: Assessing public health risk

**DOI:** 10.1016/j.heliyon.2023.e19455

**Published:** 2023-08-24

**Authors:** Md. Jahangir Sarker, Saima Sultana, Sohana Hossain, Jimmy Yu, Takaomi Arai, M. Belal Hossain

**Affiliations:** a Department of Fisheries and Marine Science, Noakhali Science and Technology University, Noakhali, 3814, Bangladesh; b School of Engineering and Built Environment, Griffith University, Brisbane, QLD, 4111, Australia; c Environmental and Life Sciences Programme, Faculty of Science, Universiti Brunei Darussalam, Jalan Tungku Link, Gadong, BE1410, Brunei Darussalam

**Keywords:** Tiger shrimp, Heavy metals, Cox's bazar, Pollution index, Public health risks, Shrimp diets, Surface sediments

## Abstract

The contamination and risk assessment of heavy metals (HMs) in highly priced tiger shrimp and its associated ecosystems and farming conditions (hatcheries and commercial grow-out ponds) were scarcely investigated in South Asian countries. In this study, we determined the five HMs (Cu, Zn, Pb, Cd, and Cr) concentrations in PL_14_ (fourteen days of Post-Larva) of *Penaeus monodon*, commercial diets, surface water, and sediments from hatcheries, farms, rivers using ICP-MS for two years. The results revealed that surface sediments of rivers and hatcheries had the highest amounts of Cr (65.85 ± 0.82 and 72.50 ± 0.42 mg/kg), Cu (18.82 ± 3.96 and 19.26 ± 4.61 mg/kg), and Zn (63.74 ± 11.14 and 87.42 ± 17.96 mg/kg), whereas commercial farms had the greatest levels of Cd (0.09 ± 0.05 mg/kg). Pb was significantly higher in sediment of hatcheries and farms than in other sites. Except for Zn in surface waters, all metals were found above the recommended limit. In case of supplied feed, all values were in the safe limit excepting Cr (3.39 ± 1.45 to 108.92 ± 3.49 mg/kg). On the other hand, among the metals, only Cr (VI) ranging from 1.75 ± 1.39 mg/kg in *P*. *monodon* samples exceeded the suggested international guidelines. The I*geo* values of all the metals were Igeo <0, indicating that the study areas were practically unpolluted. PLI values in every station were found to be below 1 which indicates the perfection of the sediment. The Potential Risk Index (PERI) values were less than 150 suggesting low risk of metals in sediments. The public health risk assessment estimated through the calculated daily intake (EDI), target hazard quotient (THQ) and hazard index (HI) has shown that the shrimp was safe for consumers except for Cd and Cr. The THQ for Cd and Cr were higher than the threshold (>1) indicating potential health hazards. The low CR values for Cd, Cr Pb were 3.1 × 10^−4^, 3.7 × 10^−4^ and 1.6 × 10^−4^, respectively indicates no cancer risks upon consuming *P. monodon*.

## 1 Introduction

Shrimp is the most commonly consumed seafood, which has ranked second in value terms after being the most-traded product for decades. These seafood items are rich in proteins, antioxidants and heavy metals such as Cu, Fe, Mn, omega-3 fatty acids, vitamin B and D [1]. These items are mainly produced in developing countries like Bangladesh, and noteworthy quantities of this production enter into international trade. This is why Bangladesh has achieved the 5th position in global crustacean production and earned the second-largest export industry that garnered about 4.7% of GDP and 9.38% of total exports (Shamsuzzaman et al., 2020). In 2020, the global shrimp production valued at US$ 18.30 Billion and is anticipated to be US$ 23.4 Billion by 2026 [1]. Despite being the world's richest ecosystems with immense productivity, the production is declining due to pollution and outbreak of shrimp disease that ultimately decreased prices and supply-demand [[1], [2]]. For example, due to supply and demand imbalances in the United States of America (USA), the European Union (EU), and Japan, shrimp prices have dropped by 15–20% in 2015 compared to 2014 [2] Furthermore, in 2009, EU governments declared a prohibition on 54 shrimp shipments from Bangladesh as a "Rapid Alert," citing food safety concerns[3].

Because of the favorable environment and space availability in Bangladesh, shrimp aquaculture has grown predominantly in low-lying tropical and subtropical coastal regions [4]. Currently, water pollution has been significantly increasing, resulting in tremendous aquatic environmental disasters[2]. Agricultural runoff, shipbreaking activities, urbanization and industrial and other anthropogenic activities in the nearshore coastal areas lead to the discharge of colossal amounts of HMs into the surface water and ultimately accumulate in the water, sediments and aquatic biota [[3], [4], [5], [6]]. It has been reported that 30–98% of HMs in rivers are transported in sediment-associated forms[7]. Besides, HMs contaminated river water enters the connected coastal areas[4], commercial shrimp farms and hatcheries [8]. In addition, the hatcheries PL and farmed *P. monodon* are fed commercially manufactured feeds for nutritional supplements and boosting growth[1,8]. For successful intensive and semi-intensive shrimp farming, quality and nutritionally balanced feeds are the prerequisites to boosting production[8]. These feeds are typically prepared with rice bran, soya meal, flour, fish meal, shrimp meal, beef liver and other ingredients but worryingly the protein materials are derived from tannery solid wastes in Bangladesh which contain HMs during the manufacturing process or from the raw materials in the feed industry[1,8].

For humans, a small amount of HMs is advised as a daily allowance (RDA as mg/day) for Zn (8.0 female, 11.0 male), Cu (1.0 kid, 10.0 adults), Cr (VI) (0.035), Pb (0.005), and Cd (0.025), respectively. Animal oxidation-reduction reactions and other biochemical and physiological processes are supported by these HMs [[9], [10]]. Atypical human metabolisms are caused by excessive HM ingestion because it interacts with diffusing ligands and macromolecules to cause bio-magnification, bioaccumulation, and integration into the marine food chain [11]. According to Tchounwou et al. [12]; Cu and Zn are both essential nutrients that support the production of hemoglobin, the metabolism of carbohydrates, and cytochrome-*c*-oxidase. However, a high consumption of these metals can increase plasma cholesterol, which can lead to coronary heart disease[13]. The forms of naturally occurring Cr range from Cr (II) to Cr (VI). Cr (VI) is mostly emitted from anthropogenic and industrial sources, and it naturally occurs in groundwater and surface water [9,12]. Cr facilitates the metabolism of glucose, but a deficiency might hinder growth and disrupt the metabolism of proteins, lipids, and carbohydrates. But in extreme circumstances, Cr(VI) can harm the lungs, liver and kidneys [12,14], as well as induce hematological, cardiovascular, respiratory, renal, gastrointestinal, and neurological dysfunctions [15]; Tore et al., 2021)). However, severe exposure can result in mental blockage, coma, or even death[16]. Chronic exposure to Pb may also lead to renal failure and liver damage[9,17]. According to Rahman et al. (2010), Cd is another contender that has the potential to harm the kidneys and result in abnormalities in infertility, kidney functioning, malignancies, hypertension, and hepatic dysfunction.

HMs concentrations and health risks in *P. monodon* have been studied in various farms and rivers in the greater Khulna and Satkhira regions[18,19]; and Sundarbans mangroves [20] (Bangladesh). HMs concentration in *P*. *indicus*, surface water and sediments in the Cox's Bazar hatcheries and Bakkhali river, Chittagong port area, Sundarbans and Meghna estuary (Bangladesh) were also studied [6]. Furthermore, HMs concentrations in seafood such as fish, shrimp, lobster, and crabs, as well as surface water and sediments, have been documented from Saint Martin Islandalong the Bay of Bengal [[21], [22], [23]]. In addition, some authors [1,8,[24], [25], [26], [27]] have documented the HMs concentration in different commercial fish and shrimp feeds in Bangladesh. HMs in water, sediments and *P*. *monodon* in the Indian Sundarbans were documented by some authors [28,29].

Renowned for its high-quality shrimp varieties, Bangladesh is one of the leading shrimp exporters' countries to Europe and America establishing itself as a significant player in the global seafood market. Tiger shrimp exports in particular make a significant contribution to the nation's foreign exchange profits, enhancing its trade balance and helping the economy as a whole. In Bangladesh, shrimp farming provides a living for around 85 million people (mainly coastal residents), is the second-largest source of foreign exchange, and accounts for about 5% of the country's GDP [19].Therefore, stringent quality control measures should be in place throughout the supply chain, ensuring that the exported shrimp meets the rigorous European Union's food safety regulations to protect consumers' health and wellbeing. Earlier studies have focused on either heavy metal in shrimp or in any component of its ecosystem[1,[24], [25], [26]]. However, a holistic assessment of heavy metals in wild and cultured shrimp, supplied feeds, and their habitats from Bangladesh and the rest of the world have meagerly been studied. Unraveling the contamination status and risk throughout the seafood supply chain can safeguard public health regionally and globally. Therefore, by employing a comprehensive and multidimensional approach, we embark on a groundbreaking study to unravel the intricate web of heavy metals present in wild and cultured shrimp (PL and tissues of *P*. *monodon)*, supplied feeds, and their associated habitats (farm, hatchery, rivers) from Bangladesh. The objectives of the present study was: (i) to measure the concentration of five metals [Pb, Cd, Cr (VI), Cu, and Zn] in water and sediment in the selected ecosystems; (ii) to evaluate the level of toxic metals in supplied shrimp feeds at farms and hatcheries; (iii) to assess the accumulation levels of metals in *P*. *monodon* PL_14_, muscle and exoskeleton/carapace samples; and (iv) to determine the probable health risk as well as the pollution status in the collected samples and ecosystems. This study advances our knowledge of the relationship between heavy metals, shrimp production, and environmental health, ultimately paving the road for a safer and reliable seafood supply for Bangladesh and beyond.

## 2 Materials and method

### 2.1 Study area and site selection

**Fig. 1:**
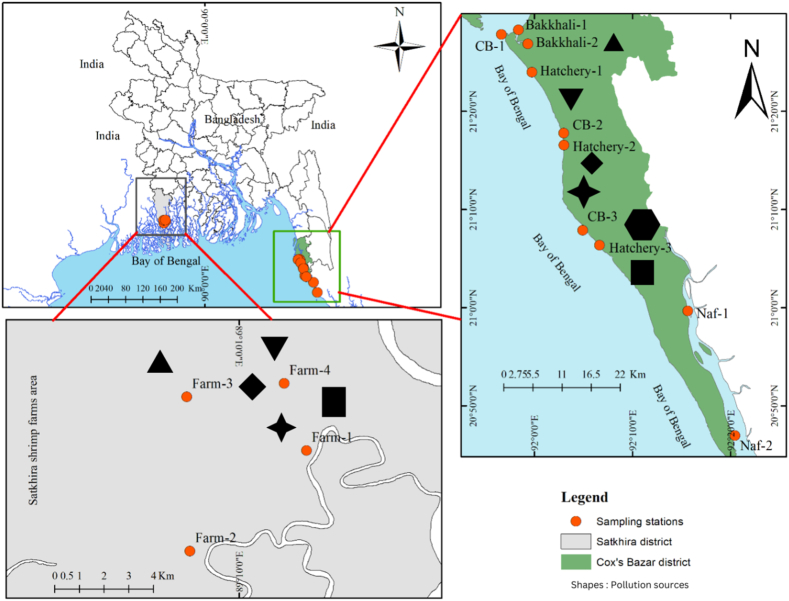
Map of Cox's Bazar district, Bangladesh where red colored circles showing sampling stations. Different shapes indicate possible pollution sources in two coastal districts (In Satkhira: Satkhira Metals, Alif feed mills, Radiant Hatchery, Tapos Agro Industry, Bismillah Weldings, and in Cox's Bazar: Ottoman Concrete Industries, Tazbir Block and Tile Industries, M/S Alam Food Industries, Akson Agro Food Industries, Niribili fish Feed Industries, Tanvir Enterprise).

Fourteen sampling sites were selected from five different ecosystems, namely: commercial hatcheries, the Bakkhali River, Naf River, the Cox's Bazar coast and commercial shrimp farms (Fig. 1). The commercial shrimp farms were selected in the Shymnagar Upazila which lies between 21°36′ and 22°24′ N latitudes, 89°00′ and 89°19′ E longitudes under the Satkhira District, Bangladesh. Approximately, 16 km area was selected near the Sundarbans mangrove. Commercial Farms 1 and 2 were chosen in the Munshiganj-Harinagar area, while Farms 3 and 4 were chosen near the Chuna River (which is directly connected to the Sundarbans mangrove rivers). During high tide, brackish water is entered (through the sluice gate) into the farms through the mangrove channel. It requires a four month *P*. *monodon* culture cycle for each harvest and yearly two crops are targeted. Before starting the culture, commercial farms are prepared by renovating the dikes, weeding and reducing silt sediments. Usually, thirteen to fifteen days post larvae (PL_13-15_) are stocked for culture and different branded commercial feeds, such as starter, grower, and finisher are fed to the PL. These PL are hatched in the hatcheries that are situated in the Cox's Bazar district.

Cox's Bazar (21.4272 N, 92.0058 E) encompasses Bangladesh's largest coastal areas as well as the world's longest unbroken natural sandy beach (125 km long). It has an unprecedented natural attraction with tertiary hills, dunes, and the vast ocean. This coastal town receives around two million tourists from home and abroad, especially from November to March. Bakkhali and Naf Rivers are one of the main gateways to the harbor which is directly connected to the Bay of Bengal in the Indian Ocean. Both of the rivers are inundated by semi-diurnal tides. In consideration of the ecological aspects of Cox's Bazar town, it is divided into two sub-sites (Bakkhali River and hatchery sites)[6]. Hatchery sites are located close to the beach areas and the Bakkhali River (opposite direction) (150–200 m).Gravid *P. mondon* are caught in the deep sea and brought to the hatchery complex for breeding purposes and >53 hatcheries actively produce commercial PL. The Bakkhali River is approximately 0.5 km wide and >8.0 m deep and it is diluted in the Moheskhali channel of Cox's Bazar. On the other hand, the Naf River is situated at the extreme south-eastern end of Bangladesh which shares the maritime boundaries between Bangladesh and Myanmar. It is about 1.6 km wide and >15 m deep. Several anthropogenic pollutants, such as garbage loads, beachside activities, restaurants, large container ships, fishing boats, trawlers, household wastes, waste from fish processing plants, plastic materials, etc. ultimately dilute the Bay of Bengal. In addition, city dwellers and tourists use these areas as local water routes for business, tourist ships bound for Saint Martin Island are harbored to the river ports. To understand the HMs pollution status and influence on popular *P. monodon*, ten sampling sites were selected, where, two sites of each in the Bakkhali (Bakkhalir-1 and 2) and Naf (Naf-1 and 2) rivers, three sites of each of the commercial shrimp hatcheries (Hatchery-1, 2 and 3), and the Cox's Bazar coast (CB-1, 2 and 3) were chosen, respectively (Fig. 1).

### 2.2 Sample collection

In total, 45 commercial shrimp feeds, 70 surface sediments, 70 water samples, and 540 *P*. *monodon* samples were collected in the past two years. Nine different feed samples (five of each) from two well-known feed companies were collected from the selected and nearby farms, and feed dealers in the Satkhira district. The collected feed samples were labeled and stored in an airtight container in the refrigerator at 4 °C.

The surface sediment samples (<5.0 cm thickness) were collected using Ekman dredge and kept in a clean polypropylene bag. At every sampling site, sediments were gathered from five places at 3.0 m intervals and approximately 300.0 g samples were sealed and marked in a polypropylene bag. Twenty samples were collected from commercial shrimp farms, ten samples from the Bakkhali and Naf rivers, and fifteen samples from the Cox's Bazar area. Furthermore, hatchery sediments were collected with a clean stainless spatula from three hatchery units and two samples in the surrounding area, as well as five samples in a hatchery. In total, 15 surface sediments were collected from the three commercial hatcheries. All the sediment samples were labeled in airtight bags and kept at 4 °C.

Likewise, the surface water samples (<100 cm depth) were collected in plastic bottles (1.0 L capacity) from the same place where the sediment samples were taken. All the bottles were previously treated with HNO_3_ (10.0%), rinsed repeatedly with deionized water and labeled properly. In total, 70 water samples were collected from the selected sites. All the samples were treated with nitric acids at pH < 2 (Merck KGaA, Germany), filtered through Whatman filter paper (0.42 μm pore size) and poured into 100 ml pre-cleaned high-density polyethylene tubes. Then the samples were stored at 4 °C for further analysis.

A 4.0 mm mesh push net was used to gently catch alive *P*. *monodon* or tiger shrmip from commercial shrimp farms, hatcheries and wild sources by local fishers. Tiger shrimp was chosen because it is one of the most economically significant and commercially lucrative shrimp species in the aquaculture sector for Bangladesh. Large size, quick growth, and strong market demand make it a popular option for the farmers and consumers. In total, 80 (20 of each) from four commercial farms, 300 fourteen days post larvae (PL_14_) (100 of each) from three commercial hatcheries, 40 from Bakkhali river, 60 from Naf river and 60 from Cox's Bazar coast of *P*. *monodon* were collected, respectively (Table 1). Immediately after harvesting, all the samples were thoroughly rinsed with deionized water and deposited in an icebox box at 4 °C. Finally, all the feed, sediment and water samples were stored at 4 °C and *P*. *monodon* was kept at −22 °C in the laboratory for HMs analysis, accordingly.

**Table 1:** The mean length and weights of *P*. *monodon* were considered in the study.

*P*. *Monodon* Sources	Mean weight ± sd (g)	Mean length ± sd (cm)	N
Hatchery PL_14_	0.014 ± 0.004	3.51 ± 0.21	300
Farms	61.55 ± 6.71	17.53 ± 3.66	80
Bakkhali river	25.13 ± 4.46	12.82 ± 3.12	40
Naf river	27.04 ± 3.81	14.11 ± 4.06	60
Cox's bazar	22.21 ± 4.44	11.08 ± 3.12	60

### 2.3 Sample preparation, digestion and metal extraction

Before analysis, the *P. monodon* samples that were kept in the lab at −22 °C and then were thawed. Using a tape measure and a digital balance (YY-768, Xpart, RFL, Bangladesh), the total body length (cm) and weight (g) were measured, respectively. The carapace and muscles were separated using forceps and a sterilized, sharp knife. A VacCo2 series freeze drier (Zirbus, Germany; condenser volume, 5.7 L; capacity, 2 kg/d) was then used to freeze-dry 1.0 g of each sample at −40 °C. All of the PL samples (100 of each) were utilized for the freeze-drying. The samples that had been freeze-dried were then put into acidic water, rinsed in a porcelain mortar, made into a fine powder with a pestle, and then frozen at −14 °C until analysis. Each freeze-dried sample was precisely weighed at 0.25 mg using a computerized electrical balance (Model: PS.P3.310, P-Scale, Taiwan). The following ingredients were prepared for digestion: 5 mL of deionized water, 2 mL of hydrogen peroxide (H2O2, 30%, Merck KGaA, Germany) 5 mL of ultra-pure nitric acid (65% HNO3, Merck KGaA, Germany). The digestion jars were then filled with the digestive reagents and weighed samples. The sample-containing vessels were then placed in microwave digestion (1000 W, Berghof-MWS2, Berghof speed wave, Eningen, Germany) by the following program after being combined for 5 min in a vortex mixer (2000 rpm, Mod. HS120214, Heathrow Scientific): 10 min at 180 °C and 800 W, then 10 min at 190 °C and 900 W, and finally 10 min at 100 °C and 400 W. After being digested, the mixture was put into a Teflon tube and filtered using Whatman paper (0.42 m pore size). 50 mL high-density polypropylene tubes (Nalgene, New York) were filled with Milli-Q water and then transferred into the tubes.

The proximate compositions of the collected feeds were recorded in the bags and leaflets provided by the manufacturing companies. Usually the commercial feeds in Bangladesh contain 30.0–36.0% crude protein, 4.0–8.0% crude lipid, 28.0–37.0% carbohydrates, 8.0–11.0% ash, 4.0–5.0% crude fiber and 9.0–12.0% moisture. In addition, comparatively, the starter feeds contain higher crude proteins and lipids than the grower, finisher and mixer feeds[8]. Precisely 0.5 g of each feed sample was added to 10 ml of ultra-pure HNO_3_ and mixed for 2 min in a vortex mixer (2000 rpm, Model: HS120214, Heathrow Scientific). Then the mixer was placed in a microwave digestion system (1000 W, Berghof-MWS2, Berghof speed wave, Eningen, Germany) for 10 min at 190 °C and adjusted to 900 W. After digestion, Milli-Q water was filled into a tube and the mixer was filtered through Whatman filter paper (0.42 μm pore size). Then the stock was transferred into 50 mL high-density polypropylene tubes (Nalgene, New York).

All of the sediment samples (70) were pulverized with a mortar and pestle and then sieved (100 m aperture) after being dried at 50 °C for 24 h (Hyun et al., 2007). Additionally, samples were dried at 60 °C for 5 h and sieved (60 m) to produce finer grinds. For digestion, 10 ml of ultra-pure HNO3 were combined with 0.5 g of each sediment sample for 5 min at 2000 rpm in a vortex mixer. Then, the mixer was put in a microwave digestion for 10 min at 190 °C with a power setting of 900 W (1000 W, Berghof-MWS2, Berghof speed wave, Eningen, Germany). After being digested, the mixture was put into a Teflon tube and filtered using Whatman paper (0.42 m pore size). High-density polypropylene 50 mL tubes (Nalgene, New York) were filled with Milli-Q water and then transferred into them.

### 2.4 Analysis of samples

ICP-MS (ELAN 9000, PerkinElmer, Germany) was used to examine each sample. An ELAN 9000/6X00, TruQTMms, Germany, multi-component standard solution was used to standardize the calibration. The calibration solution (20.0 g/mL: Cd, Cu, Pb, Mg, Rh, 1% HNO3) purchased from PerkinElmer was used to confirm the relative standard deviation (RSD of 10%) before the analysis started. Pb = 0.001, Cd = 0.0005, Cr = 0.003, Cu = 0.01 and Zn = 0.03 mg/kg, respectively, were chosen as the limits of detection (LOD). Additionally, PerkinElmer sold internal calibration standard solutions with 0.5 mg/kg of indium (In), yttrium (Y), cobalt (Co), and thallium (TI) in each case. The multi-component stock Merck solution, 1000 mg/L (Merck KGaA, Germany), was diluted to provide the working standards (0, 0.2, 1.0, 5.0, 10, 20, 50, and 100 g/L). Only if the value met the specified internal calibration point was a test batch counted. One blank sample and one authenticated reference material, NMIJ CRM 7402-Cod fish tissue assessed by ICP-MS (mean SD, mg/kg, dw), were utilized for each batch analysis. In order to prevent batch-specific mistakes, a few specimens were measured in triplicate. For the chosen HMs, the highest average recoveries (%) were recorded between 96.7 and 110.

**2.5. Ecological and public health assessment:** Pollution load index (PLI), Geo-accumulation index (Igeo), Potential ecological risk index (RI), Estimated daily intake (EDI), Target hazard quotient (THQ), Carcinogenic risk (CR) were calculated using the equations presented in Table S1.

### 2.5 Statistical analysis

The univariate and multivariate statistical analyses were carried out using the free and open-source PAST software (version 4.02). The calculated HMs in sediment, water, feed and shrimp were presented as mean ± SD. In the cited literature and guidelines HMs concentration that was measured in wet weight (wt.) was converted into dry wt. by assuming an average of 74% water present in tissues and presented within brackets as dry. wt. mg/kg.

## 3 Results and discussion

### 3.1 HMs concentration in water

HMs concentration in water from different ecosystems has been presented in Table 2. Pb, Cd, Cr (VI), Cu and Zn ranged from 0.01 ± 0.002 to 0.04 ± 0.03, 0.007 ± 0.001 to 0.04 ± 0.01, 0.09 ± 0.01 to 0.49 ± 0.22, 0.03 ± 0.02 to 0.43 ± 0.47, 0.04 ± 0.02 to 0.40 ± 0.33 mg/L, respectively. Except for Zn (0.19 ± 0.05 mg/l) the measured quantities of Pb (0.02 ± 0.02 mg/l), Cd (0.013 ± 0.012 mg/l), Cr 0.18 ± 0.16 mg/l), and Cu (0.27 ± 0.11 mg/l) in the surface water in the examined habitats were greater than the suggested limit. The higher concentration of detected metals in water resources of the studied habitat was mainly due to various environmental instabilities; caused by effluent discharge from industries and factories (cement industries, metal industries and agrofood based farms), as well as direct disposal of sewage and solid waste into rivers and coastal regions [30]. In addition, farms and hatcheries receive additional metal loads from commercial shrimp feeds. Different human activities and natural processes contribute to heavy metal pollution in the water of coastal locations. Metal contamination in Cox's Bazar is a result of industrial effluents being discharged into waterways untreated or insufficiently treated. Heavy metals may be dissolved into rivers through runoff after rainfall as a result of excessive fertilizer and pesticide use in agro-farms. Once more, increased urbanization and poor waste management in cities can cause heavy metals to be dumped directly into rivers or to be carried there by storm water runoff. Weak waste management procedures and insufficient enforcement of environmental laws may result in the careless dumping of garbage containing heavy metals, raising the possibility of contamination. Natural weathering and erosion of rocks and minerals can contribute to the presence of heavy metals in river water. However, HMs concentrations in the surface water of the Bakkhali estuary and shrimp hatcheries in Cox's Bazar did not exceed the recommended guidelines in comparison to our study [6].

**Table 2:** Heavy metals in water of different ecosystems of shrimp. (n = 70, mean ± SD, standard deviation).

Sites	Pb	Cd	Cr	Cu	Zn	References
Hatchery (Mean)	0.02 ± 0.01	0.01 ± 0.004	0.09 ± 0.11	0.14 ± 0.03	0.13 ± 0.16	Present study
River (Mean)	0.02 ± 0.002	0.02 ± 0.002	0.29 ± 0.16	0.57 ± 0.11	0.26 ± 0.05	
Cox's Bazar (Mean)	0.02 ± 0.02	0.009 ± 0.001	0.21 ± 0.43	0.25 ± 0.11	0.30 ± 0.09	
Shrimp Farm (Mean)	0.03 ± 0.02	0.01 ± 0.01	0.14 ± 0.21	0.12 ± 0.21	0.07 ± 0.23	
Meghna River,	0.01	0.018	0.020	0.027	0.040	[31]
Bakkhali estuary	0.004	0.000	0.003	0.239	0.239	
Cox's Bazar Hatchery	0.11	0.000	0.009	0.51	0.51	[6]
USEPA, WHO	0.01–0.05	0.003–0.01	0.050–0.1	0.05–2.0	3.0	[32]; [33]

### 3.2 HMS concentrations in sediment

**Fig. 2:**
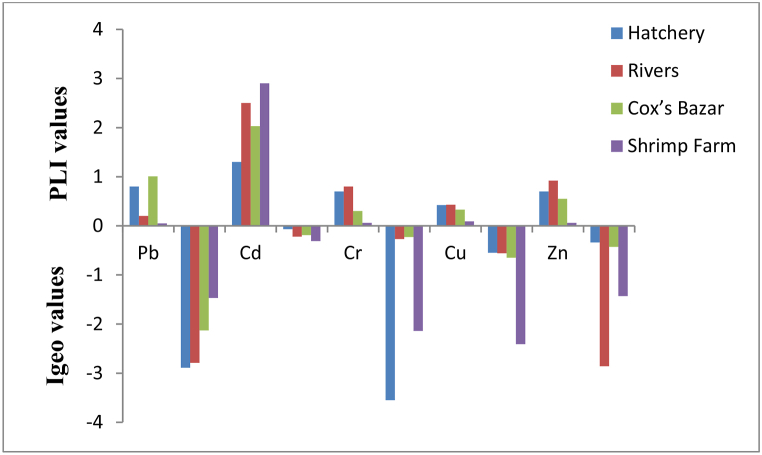
Pollution related hazard indices (PLI and Igeo) for metal concentrations in sediment.

The studied sediment samples from different habitats included a wide range of heavy-metal concentrations (Table 3). The loads of five selected heavy metals in the sediment of rivers and farms followed the decreasing order of: Zn (87.42 ± 17.96) > Cr (72.50 ± 0.42) > Cu (19.26 ± 4.61) > Cd (0.08 ± 0.02) > Pb (0.05 ± 0.05) and Cr (6.13 ± 2.22) > Zn (5.24 ± 2.18) > Cu (4.27 ± 2.48) > Pb (1.05 ± 0.46) > Cd (0.09 ± 0.05), respectively. In addition, HMs concentrations in the sediments of hatcheries and Cox's Bazar coasts followed as follows: Cr (65.85 ± 0.82) > Zn (63.74 ± 11.14) > Cu (18.82 ± 3.96) > Pb (1.50 ± 0.21) > Cd (0.04 ± 0.01) and Zn (51.88 ± 30.71) > Cr (23.17 ± 0.42) > Cu (14.64 ± 11.83) > Cd (0.06 ± 0.04) > Pb (0.01 ± 0.001), respectively. The highest and lowest average HMs concentrations in sediments were measured in rivers (35.860 mg/kg) and commercial shrimp farms (3.35 mg/kg), respectively. These rivers are directly linked to human settlement, regular touring activities, industrial effluents (Agrobased farms) etc. that discharge untreated or poorly treated waste [[6], [34],^35^]. On the other hand, commercial farms in the Shymnagar Upazila are isolated from the industrial zone and situated between the Bangladesh and India borders. Moreover, the farm dikes are annually renovated by the surface sediments of the farms which may result in lower HMs concentrations. Nevertheless, HMs intensity in the shrimp farm sediment might be influenced by shrimp feeds which naturally contain HMs in the soil and coastal water[36]. HMs like Pb, Cd, Cr and Cu ranged from 0.921 to 1.058, 0.122–0.160, 1.957–3.436, and 0.801–1.176 mg/kg, respectively in shrimp farms in the Satkhira district, Bangladesh[37]. However, HMs in the Mymensingh fish farm (Bangladesh) sediments were enormously higher than our measured values and ranged from 11.5 to 18.5, 0.007–0.011, 53.5–77.3, and 100–250 mg/kg in Pb, Cd, Cr and Zn, respectively [38]. The average HMs concentration was 30.0 mg/kg in commercial hatcheries and 18.0 mg/kg on the Cox's Bazar coast. Both sites are close to one another in terms of ecology, and the hatchery units use coastal seawater for breeding and raising. Furthermore, given that anthropogenic activities and nearshore industry both have an impact on these ecosystems, semipermeable HMs access may exist between them [6]. Furthermore, the average HMs concentrations in the hatcheries were determined to be higher than the Cox's Bazar coasts. It is possibly due to the HMs contents in the commercial feeds provided to the hatchlings and rearing PL, using ZnO for oxygen supply, improper handling, direct contact with HMs containing materials, or transmission from their parents [6]. In the Cox's Bazar hatcheries, Pb concentrations ranged from 6.2 to 38.9 mg/kg which was more than twentyfold higher than our estimated values. However, the Cd, Cr, Cu and Zn concentrations ranged from 0.003 to 0.004, 10.7–18.0, 2.4–4.3, and 15.4–24.8 mg/kg in the selected hatcheries in Cox's Bazar which were lower than our determined values (Table 3; [6]. In the coastal sediments of the Bay of Bengal Cd (2.8–6.1 mg/kg) was determined to be more than five times higher than our estimated values in the selected ecosystems [22]. We determined higher HMs concentrations in the Bakkhali and Naf rivers than the hatcheries that were similarly reported by [6] in the Bakkhali river estuary. Furthermore, the HMs content in the Naf River was higher than its nearby coastal areas like Teknaf Port and Saint Martin's Island of the Bay of Bengal[34]. Pb concentrations in the Teknaf port, Naf river, and Saint Martin's Island coasts ranged from 10.0 to 37.5 mg/kg, which was more than twenty times higher than our measured values (Table 3). Although Cr and Cu concentrations exceeded the guidelines by CB-TEC and TEL in hatcheries and rivers, it was within the limits for CB-PECs, PEL and PEL- EC. Nevertheless, Pb, Cd, Cu and Zn did not exceed the provided guidelines (Table 3). The Igeo values of all the metals remained below zero (Fig. 2), indicating that the study areas are practically unpolluted (Igeo <0). For assessing the temporal contamination history, PLI values for all the samples were calculated (considering the contribution of all elements) to understand the combined heavy metal loads. The sediment samples showed low to moderate contamination, with PI values ranging from 0.05 to 2.9 (Fig. 2). However, PLI and RI values were <1.0 and < 150, respectively; indicated that the selected HMs showed weak individual potential risks no pollution load index (PLI) and lower potential ecological risk index (RI). PLI values in every station were found to be below 1 which indicates the perfection of the sediment ( ( Fig. 2).

**Table 3:** HMs concentration in surface sediments in different ecosystems in Bangladesh along with relevant literature and guidelines. (n = 70, mean ± SD, standard deviation).

Study area	Pb (mg/kg)	Cd (mg/kg)	Cr (mg/kg)	Cu (mg/kg)	Zn (mg/kg)	References
Hatchery (Mean)	1.50 ± 0.21	0.04 ± 0.01	65.85 ± 0.82	18.82 ± 3.96	63.74 ± 11.14	Present study
Rivers (Mean)	0.05 ± 0.05	0.08 ± 0.02	72.49 ± 0.42	19.26 ± 4.61	87.42 ± 17.96	
Cox's Bazar (Mean)	0.01 ± 0.001	0.06 ± 0.04	23.17 ± 0.42	14.64 ± 11.83	51.88 ± 30.71	
Shrimp Farm (Mean)	1.05 ± 0.46	0.09 ± 0.05	6.13 ± 2.22	4.27 ± 2.48	5.24 ± 2.18	
Relevant literature						
Halda River	8.80	0.04	8.84	5.90	79.58	[39]
Fish farm	208 ± 31.388	0.009 ± 0.001	63.054 ± 6.922	NA	208 ± 31.388	[38]
Shrimp Farms	0.921–1.113	0.122–0.160	1.957–3.436	0.801–1.176	NA	[37]
Turag River	32.78 ± 3.32	0.28 ± 0.33	43.02 ± 18.31	50.40 ± 5.62	139.48 ± 42.48	[40]
Passur River	5.33–18.42	0.80–2.70	2.80–31.90	11.48–29.25	26.25–71.93	
Ganga (India)	10.94–44.89	0.94–2.86	39.05–93.28	12.71–36.68	41.05–92.48	[3]
Jiuzhen Bay (China)	136	0.062	NA	8.6	57	[41]
Bohai and Yellow sea (China)	26.79 ± 11.35	0.160 ± 0.189	56.80 ± 20.29	22.07 ± 12.72	77.98 ± 51.54	[42]
Red sea (Saudi Arabia)	3.86 ± 0.82	0.48 ± 0.12	5.64 ± 1.51	16.39 ± 3.81	24.74 ± 6.47	[43]
Fish Farms Egypt (El-Fayoum Province)	0.07	0.006	NA	0.04	0.75	[44]
Background values						
^1^Bakkhali river	24.47	0.02	24.33	13.3	36.7	[6]
^2^Shale	20	0.3	90	45	95	[45]
^3^Naf river	23	0.6	17	16.5	70.3	[[23], [34]]
^4^Cox's Bazar	12.64	0.85	30.51	30.28	84.56	[[6], [23]] (2015); [34]
Guidelines						
^5^CB-PECs	128	4.98	111	149	459	
^6^PEL	91.3	3.53	90	197	315	[46]
^7^CB-TEC	35.8	0.99	43.4	31.6	121	
^8^TEL	30.2	0.68	52.3	18.7	124	[46]
^9^EC-PEL	112	4.21	160	108	271	[47]

^1^The background values of Bakkhali river was cited from Ref. [6]; ^2^Due to having no reliable data, Shale was cited as background values for hatchery and shrimp farms from Ref. [45]. ^3^The background values of Naf river was cited from Refs. [23,34]. ^4^The of background values Cox's Bazar was cited from Refs. [23,6,34]. ^5^Consensus-based probable effect concentrations (CB-PECs); ^6^PEL- Probable effects level; ^7^CB-TEC (Threshold effects concentration); ^8^TEL- Threshold effects level; ^9^EC- Environment of Canada-PEL. Below the detectable limit (BDL) and Not analyzed (NA).

### 3.3 HMs concentration in shrimp feeds

HMs were assessed from nine commercial shrimp feeds and the values ranged from 0.06 ± 0.034 to 0.14 ± 0.07, 0.006 ± 0.004 to 0.08 ± 0.07, 3.39 ± 1.45 to 108.92 ± 3.49, 0.63 ± 0.001 to 1.74 ± 0.11 and 0.23 ± 0.11 to 0.08 ± 0.05 mg/kg, in Pb, Cd, Cr (VI), Cu and Zn respectively. HMs in the analyzed feeds were lower than the Bangladesh standard, except for Cr (VI) which exceeded both national and international standards (Table 4). Comparatively, the shrimp feeds had higher HMs than fish feeds and Pb exceeded both the national (0.5 mg/kg) and international (2.0 mg/kg) guidelines (Table 4). The higher Pb (4.45 ± 0.02 mg/kg) and Cd (2.20 ± 0.97 mg/kg) contents were measured in the Feed-A (Bangladeshi) that crossed both of the guidelines[1]. In addition, the HMs concentration was measured in Feed-B (Chinese) which exceeded all the provided guidelines[48]. This is due to shrimp feed include rice bran, wheat, fish, shrimp, soy, beef liver, silkworms, and earthworms that contain substantial levels of heavy metals [1]. In the fish feeds manufactured by some well-known companies of our country, Cr concentrations were measured at 8.21 mg/kg and 15.55 mg/kg, respectively[25]. Furthermore, the Cr ranged from 20.25 to 301.5 mg/kg in the starter, grower, and nursery fish feeds formulated by another feed company of Bangladesh[24].

**Table 4:** HMs concentration (mg/kg, dw) in shrimp feeds, comparison with other literature and guidelines. (n = 45, mean ± SD, standard deviation).

Brand/country	Feed type	Pb	Cd	Cr	Cu	Zn	References
**Feed-A** **Bangladesh**	PL Starter	0.06 ± 0.04	0.008 ± 0.005	7.56 ± 0.53	0.80 ± 0.005	0.15 ± 0.06	
	Starter crumble	0.06 ± 0.04	0.01 ± 0.006	10.13 ± 4.01	0.80 ± 0.005	0.14 ± 0.03	
	Nursery	0.06 ± 0.05	0.02 ± 0.01	4.67 ± 1.07	0.72 ± 0.006	0.17 ± 0.05	Present
	Grower	0.06 ± 0.03	0.009 ± 0.005	3.39 ± 1.45	0.71 ± 0.006	0.08 ± 0.05	study
**Feed-B** **China**	Pre starter	0.16 ± 0.07	0.01 ± 0.01	14.76 ± 1.95	0.83 ± 0.006	0.23 ± 0.11	
	Starter	0.07 ± 0.005	0.006 ± 0.004	20.16 ± 0.20	0.63 ± 0.001	0.09 ± 0.004	
	Nursery	0.11 ± 0.07	0.08 ± 0.07	108.9 ± 3.49	0.82 ± 0.004	0.22 ± 0.08	
	Grower	0.14 ± 0.07	0.05 ± 0.06	94.23 ± 3.98	1.67 ± 0.10	0.14 ± 0.05	
	Finisher	0.12 ± 0.05	0.02 ± 0.02	74.16 ± 2.31	1.74 ± 0.11	0.17 ± 0.06	
	Mean	0.09 ± 0.05	0.02 ± 0.02	37.56 ± 2.11	0.96 ± 0.03	0.15 ± 0.06	
**Relevant literature**							
	Shrimp	4.45 ± 0.020	2.20 ± 0.97	<0.54	NA	NA	
	Shrimp	3.90 ± 0.020	0.17 ± 0.04	<0.54	NA	NA	
	Shrimp	4.33 ± 0.010	0.65 ± 0.151	<0.54	NA	NA	
	Shrimp	4.56 ± 0.030	1.36 ± 0.57	<0.54	NA	NA	Islam et al.(
	Shrimp	4.04 ± 0.020	0.46 ± 0.15	<0.54	NA	NA	2017)
	Shrimp	4.39 ± 0.010	0.47 ± 0.27	<0.54	NA	NA	
	Shrimp	4.44 ± 0.010	0.50 ± 0.18	<0.54	NA	NA	
	Fish feed	0.67	0.12	0.0	8.21	NA	Fatema et
	Fish feed	0.0	0.26	1.04	15.55	NA	al. (2019)
	Fish feed	0.10–2.00	0.250–1.730	NA	15.10–60.40	81.70–242.9	[48]
**Guidelines**							
	Fish feed	2.00	1.00	0.05	10.00	150.00	[49]
	Shrimp feed	0.50	0.50	0.10	5.00	50.00	
	Fish feed	0.30	0.05	0.10	5.00	50.00	[50]

### 3.4 HMs concentration in *P. monodon*

The average HMs concentration in the *P. monodon* samples were 0.02 ± 0.01, 0.02 ± 0.009, 1.75 ± 1.39, 20.95 ± 2.33 and 78.87 ± 15.67 mg/kg in Pb, Cd, Cr, Cu and Zn, respectively (Table 5). None of the estimated values exceeded the suggested guidelines, except Cr which crossed guidelines (0.40–1.93 mg/kg) by WHO (1990), [32]. In the areas of environmental protection, food safety, and public health, following guidelines published by renowned organizations like the US Environmental Protection Agency (USEPA), Food and Agriculture Organization (FAO), and World Health Organization (WHO) has several benefits. For instance, the standards established by these organizations are supported by in-depth study of data and scientific proof. They go through a rigorous evaluation process by professionals in the relevant industries, ensuring the values are precise and trustworthy. These numbers offer a reliable foundation for comparisons between various nations and areas. This uniformity helps worldwide environmental and food quality assessments as well as cross-border collaboration and trade. Adopting existing standards can improve country-to-country harmonization and streamline the regulatory process, which is advantageous for trade and cooperation. HMs accumulated in tissues like muscles, carapace and PL14. The *P. monodon* PL14 and carapace had higher HMs concentration than the muscles. Differences among metal levels in shrimp were evident in the exoskeleton and muscles. The farmed shrimp accumulated more Pb, Cd and Cr than the wild shrimp. Due to several aspects of their feeding and growing procedures, farmed shrimp may collect more heavy metals than wild shrimp[19]. For example, the diets used to feed farmed shrimp frequently include seafood that was obtained in the wild or heavy metals. These fish-based feeds have the potential to introduce heavy metals that the prey fish had acquired into the diet of the farmed shrimp. Wild shrimp, in contrast, have a more varied diet and experience varying levels of exposure to heavy metals depending on their environment. Shrimp farms can occasionally be found adjacent to commercial and industrial sectors, metropolitan areas, or areas where there are large pollution sources. In intensive shrimp farming methods, waste materials and uneaten feed can accumulate in the sediment, potentially producing heavy metal pollution of the sediment. In such settings, farmed shrimp may consume polluted sediment particles. Because of this, farmed shrimp may be exposed to higher levels of heavy metals found in water, sediment, or other environmental inputs. On the contrary, both farmed and wild shrimp accumulated parallel amounts of Cu and Zn. Similarly, the HMs concentrations were higher in *P. monodon* carapace shell than muscles reported from the Saint Martin's Island of the Bay of Bengal, and rivers and the commercial farms situated in the greater Khulna regions, Bangladesh, respectively[19].

**Table 5:** Concentration (mg/kg dry weight) of heavy metals assessed in *P. monodon* from different ecosystem in Cox's Bazar, Bangladesh along with the guidelines and literature review. (n = 540, mean ± SD, standard deviation).

	*P*. *monodon*	Pb	Cd	Cr	Cu	Zn	References
Mean value from Bakkhali River,	Hatchery PL_14_	0.03 ± 0.02	0.02 ± 0.006	3.33 ± 0.43	15.07 ± 1.08	45.99 ± 1.51	Present study
Naf River, Cox's Bazar	Farm-muscle	0.03 ± 0.032	0.02 ± 0.026	0.30 ± 0.21	20.68 ± 1.62	70.53 ± 1.89	
Satkhira shrimp	Farm-carapace	0.02 ± 0.004	0.02 ± 0.022	2.87 ± 0.44	22.06 ± 1.07	94.83 ± 2.38	
farms	Wild- muscle	0.02 ± 0.005	0.01 ± 0.004	0.19 ± 0.08	22.79 ± 1.11	74.46 ± 2.46	
	Wild-carapace	0.02 ± 0.006	0.01 ± 0.006	2.79 ± 0.33	21.22 ± 1.57	92.89 ± 2.76	
Cox's Bazar (Farm)	Muscle	17.75 ± 1.5	0.09 ± 0.03	0.69 ± 0.6	9.43 ± 2.8	18.89 ± 2.9	[13]
Bay of Bengal	Muscle	0.8–1.3	0.2–0.3	1.7–2.9	12.2–21.3	24.2–35.7	[30]
(Saint Martin Island)	Carapace	2.1–3.1	0.3–0.6	2.6–4.1	62.8–71.3	76.2–108.2	
Botiaghata (Farm),	Muscle	1.16 ± 0.1	0.08 ± 0.01	0.68 ± 0.02	NA	NA	[19]
Khulna	Shell	1.23 ± 0.11	0.12 ± 0.04	1.03 ± 0.01	NA	NA	
Rupsha river,	Muscle	0.46 ± 0.18	0.05 ± 0.01	0.15 ± 0.02	NA	NA	
Sabah, North Borneo (Farm)	Muscle	0.38–0.44	0.05	0.04–0.05	NA	NA	[51]
Gangetic delta	Muscle	9.2	7.7		11.1–48.1	16.1–447.5	[52]
Guidelines	WHO/FAO, USEPA	2	1	1	30	100	[[33], [49]]

Accumulation of heavy metals in shrimp can be considerably influenced by their size, sex, molt cycle, reductive condition and feeding behavior. For example, shrimp of the same species that are larger tend to acquire more heavy metals than shrimp that are smaller. Smaller shrimp, on the other hand, could collect low amounts of heavy metals as a result of their shorter lifespans, decreased prey consumption, and lower positions in the food chain. Again, larger shrimp eat more than smaller ones, thus they are exposed to higher concentrations of heavy metals in their diet. They accumulate heavy metals more quickly in their tissues if the food they eat is tainted with the metals. The amounts of metal in shrimp are likewise affected by molt cycles. Shrimp undergo periodic molting, where they shed their exoskeleton to grow larger. During this process, they may release heavy metals accumulated in their old exoskeleton back into the water[19]. Larger shrimp that molt less frequently have a higher chance of retaining accumulated heavy metals in their bodies. Further, male and female shrimp have different hormonal profiles, which can influence their physiology and metabolism. These differences may affect how heavy metals are taken up, distributed, and eliminated in their bodies. Other factors, such as species-specific biology, environmental conditions, and the type and concentration of heavy metals in the environment, also play significant roles in determining metal accumulation in shrimp.

### 3.5 Public health risk assessment

**Fig. 3:**
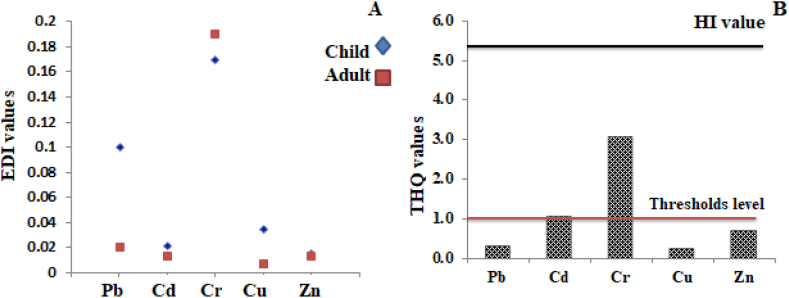
Public-health-related hazard indices (A)EDI, (B)THQ & HI for metal concentrations in shrimp samples.

To ensure consumer safety, daily intake (EDI), non-carcinogenic risks (THQ), carcinogenic risks (CR) and hazard index (HI) in *P. monodon* tissues were calculated (3A&B). Except for Cr, our calculated EDI in children was higher than that in adults. All the values are below RDA standards point indicating a potential reduction in the elements' negative effects on consumer health. Furthermore, the THQ values of Pb, Cu and Zn were within the threshold limit except for Cd and Cr which were higher than the threshold (>1) indicating potential health hazards through Cd and Cr contamination. The CR values for Cd, Cr and Pb 3.1 × 10^−4^, 3.7 × 10^−4^ and 1.6 × 10^−4^, respectively were within the acceptable range that indicates no cancer risks upon eating *P. monodon* (Fig. 3).

## 4 Conclusion

Five HMs (Pb, Cd, Cr, Cu and Zn) concentrations, distributions, pollution levels, public health and ecological risks were determined in *P. monodon* PL14, tissues, feeds, surface seawater and sediments in the greater Cox's Bazar and Satkhira regions of Bangladesh. Among the assessed HMs, Cr (VI) exceeded the suggested guidelines in *P. monodon* PL14, exoskeleton/carapace samples, and shrimp feeds. The higher Cr contents in the ecosystems could be correlated with the contaminated feed ingredients, manufacturing processes and industrial establishments. Although the TEL, PEL and EC-PEL in sediments were within the SQGs.Nevertheless, *P. monodon* could be eaten by the Bangladeshi consumers as EDI values were below RDA standards point. The health risks index (THQ and HI) were below the safety limits except for Cd and Cr contamination. Carcinogenic risks are not likely to occur as a result of heavy metals through consumption of shrimp since they were within permissible limit. Pollution load index (PLI), and Geo-accumulation Index (Igeo) showed minimal levels of contamination in the studied sites. All heavy metals do not have the same bioavailability or harm to humans or other creatures. A metal's speciation (chemical form) can have a substantial impact on its toxicity and accumulation in shrimp, but it can be challenging to take this complexity into account in studies like this one. Additionally, a number of elements, including habitat conditions and shrimp characteristics like age, size, and sex, can affect the accumulation of heavy metals. It is difficult to account for every possible confounding variable. Studies on heavy metals in shrimp and their ecosystems are nevertheless vital for understanding environmental health and guaranteeing the safety of seafood for human consumption despite these constraints. Robust study design, standardized procedures, and cooperation between researchers and policymakers can address these shortcomings and produce more accurate and thorough understandings of the contamination status or effects of heavy metals in or on shrimp and their habitats.

## Author contribution statement

Conceived and designed the experiments: M.J.S;

Analyzed and interpreted the data; M.B. H; S.S.E &S.H.A; J. Y.; T. A.

Contributed reagents, materials, analysis tools or data; M.J.S;

Wrote the paper: M.J.S.; M.B.H.; J.Y.; T.A.

## Funding statement

This study was partially funded by Universiti Brunei Darussalam under the FOS Allied Fund (UBD/RSCH/1.4/FICBF(a)/2023).

## Data availability statement

Data will be made available on request.

## Declaration of competing interest

The authors declare that they have no known competing financial interests or personal relationships that could have appeared to influence the work reported in this paper.
