# Supplementary material for: Heavy metals in wild and cultured shrimp, supplied feeds, and their habitats: Assessing public health risk
Source: Heliyon. 2023 Aug 24;9(9):e19455. doi: 10.1016/j.heliyon.2023.e19455 (PMC10481285; doi:10.1016/j.heliyon.2023.e19455)
Supplement: Multimedia component 1 [file mmc1.docx]

| Supplementary table  Table S1. Assessment of the health risk and pollution status of heavy metals in shrimp from Bangladesh coastal area | | | | | |
| --- | --- | --- | --- | --- | --- |
| Eq. No | Index | Depiction and objectives | Principle | Explanation | Pollution degree criteria |
| 2. | Estimated daily intake (EDI) | The estimated daily intakes (EDI) for the analysed metals were calculated by multiplying the respective mean concentration of the metal determined in the targeted fish samples by the weight of fish consumed by an average individual in Bangladesh | EDI = ( DFC$\times$MC)/BW | Here, DFC is daily food (fish) consumption, MC is mean metal concentration of metal in shrimp sample. The daily fish consumption rate for an adult (60 kg) was an average of 49.5 g on fresh weight basis | The recommended daily allowance guidelines by WHO |
| 3. | Pollution Load Index (PLI) | PLI can be used for assessing of comparison of contamination status among the study sites. | PLI = n√ (CF_1_ × CF_2_ × CF_3_ × ........ CFn) | Here, n is the total number of studied metals, and CF is calculated as described in the earlier equation (Eq. 1). | PLI<1: No pollution;  PLI>1: Polluted |
| 4. | Geo-accumulation Index (I_geo_) | I_geo_ is helpful in evaluating the contamination status of the environment by comparing it with geochemical background concentrations (Muller,1969) | $I_{geo}=\log_{2}\left( \frac{\mathrm{Cn}}{1.5Bn} \right)$ | Here, C_n_ is the concentration of the metals observed in sediment samples. B_n_ is the geochemical background value of the metal (n), the factor 1.5 means the possible variations in the background values (Ke et al., 2017). | <0: Practically unpolluted;  0-1: Unpolluted to moderately polluted;  1-2: Moderately polluted;  2-3: Moderately to heavily polluted;  3-4: Heavily polluted;  4-5: Heavily to extremely polluted;  >5: Extremely polluted. |
| 6. | The Potential ecological risk index (PERI) | PERI was proposed by Hakanson (1980) and is applied to appraise the potential ecological risk of studied metals in sediment. This index provides a better evaluation of the potential ecological risk factor of heavy metal contamination by combining ecological and environmental effects with toxicology (Ke et al., 2017). | $E_{r}^{i}=T_{r}^{i}C_{f}^{i}$  $C_{f}^{i}= C_{n}^{i}/C_{o}^{i}$  $ER_{I}=\sum E_{r}^{i}$ . | is the potential ecological risk factor and is the toxic response factor of studied metals. It was determined for Cu=Pb=Ni=Co=5, Mn=Zn=1, As=10, and Cr=2 (Suresh et al.,2011). | Er < 40: Low ecological risk  Er ≤80: moderate ecological risk;  80<Er≤160: appreciable ecological risk;  160 < Er ≤320: high ecological risk; Er>320: serious ecological risk  And  RI <150: Low pollution;  150<RI<300: Considerable pollution;  300<RI<600: High pollution;  RI ≥600: Very high pollution |
| 7. | Target Hazard Quotient (THQ)  and Hazard index (HI) | THQ is the non-carcinogenic risks measured by the ratio of CDI and reference dose (RfD).  The hazard index from THQs is denoted as the total of the hazard quotients by multiple elements (Pb, Cd, Cr etc.) | $THQ=\frac{MC \times IR \times EF \times ED \times CF}{\mathrm{RfD}\times\mathrm{BW}\times\mathrm{ATn}}\times$10^-3^  $\mathrm{HI}=\sum_{i=k}^{n} THQ$ | Here, MC is the heavy metal concentration, EF is the exposure frequency (365 days/year), ED is the exposure duration (30 years or 10950 days) for non-carcinogenic risk, RfD is the reference dose of individual metal, BW is an average adult body weight (70 kg) and ATn is the average exposure time for non-carcinogens (10,950 days) | If the THQ < 1, the exposed individual is unlikely to experience adverse health effects; for THQ ≥ 1, there could be a likelihood of possible health hazards.  When HI value is higher than 10, the non-carcinogenic risk effect depicts high risk for its consumers. |
| 8. | Carcinogenic risk (CR) | Carcinogenic risk (CR) indicates an incremental probability of an individual of developing cancer over a lifetime due to exposure to a potential carcinogen | CR = CSF × EDI | Here, CSF is the carcinogenic slope factor which is 0.0085 (mg/kg/day) for Pb and 1.5 (mg/kg/day) for As set by USPEA | Acceptable risk levels for carcinogens range from 10^-4^ to 10^-6^ |
